# Effect of a Mindfulness-Based Intervention for Chronic Migraine and High Frequency Episodic Migraine in Adolescents: A Pilot Single-Arm Open-Label Study

**DOI:** 10.3390/ijerph182211739

**Published:** 2021-11-09

**Authors:** Licia Grazzi, Eleonora Grignani, Alberto Raggi, Paul Rizzoli, Erika Guastafierro

**Affiliations:** 1 Dipartimento Neuroalgologia Centro Cefalee, Fondazione IRRCS Istituto Neurologico Carlo Besta, 20133 Milano, Italy; eleonora@grignani.net; 2 UO Neurologia Salute Pubblica e Disabilità, Fondazione IRRCS Istituto Neurologico Carlo Besta, 20133 Milano, Italy; alberto.raggi@istituto-besta.it (A.R.); erika.guastafierro@istituto-besta.it (E.G.); 3 John Graham Headache Center, Brigham and Women’s Faulkner Hospital, Harvard Medical School, Boston, MA 02115, USA; prizzoli@bwh.harvard.edu

**Keywords:** headache, mindfulness, adolescents, chronic migraine, disability, anxiety, depression, catastrophizing

## Abstract

In this single-arm pilot open-label study we examined the effect of a mindfulness-based intervention on reduction of headache frequency after 12 months in adolescents aged 12–18 with chronic or high-frequency migraine without aura. Adolescents were recruited at the headache center of the C. Besta Neurological Institute and followed-up for 12 months. The mindfulness-based intervention was delivered in small groups and consisted of six weekly group sessions of guided meditation, and one booster session 15 days after. Patients filled in questionnaires assessing headache frequency (primary endpoint), medication intake, disability, anxiety, depression, catastrophizing, and caregivers’ burden. Within-person ANOVA was used to address variation of endpoints over time. Thirty-five out of 37 patients completed the study for primary endpoints, and 33 for secondary endpoints. Headache frequency dropped from 21.3 (95% CI 18.5; 24.1) to 9.6 (95% CI 6.1; 13.1) days per month at 12 months (F = 30.5, *p* < 0.001); 23 patients out of 35 (65.7%) achieved a headache frequency reduction greater than or equal to 50%. Significant improvements were also reported for medication intake (F = 18.7, *p* < 0.001), disability (F = 3.8, *p* = 0.027), trait anxiety (F = 5.1, *p* = 0.009), symptoms of depression (F = 9.5, *p* < 0.001), and catastrophizing (F = 23.6, *p* < 0.001). In conclusions, our study shows a reduction of headache attacks in adolescents who follow a mindfulness-based program, suggesting benefit of this nonpharmacological approach.

## 1. Introduction

Chronic migraine (CM) is a highly disabling condition in both adults and adolescents, among whom the prevalence is around 2% [1,2,3,4,5]. It is characterized by at least 15 headache days per month, and it is often associated with the overuse of symptomatic drugs [6]. High frequency episodic migraine (HFEM) without aura, a condition characterized by 8–14 days with migraine per month [6,7], can be problematic as well because it predisposes patients to chronic migraine and medication overuse. CM and HFEM impact patients’ emotional, social, and school functioning [8,9,10,11]. A recent report showed that structured and tailored public health policies and strategies, including diagnosis, pharmacological and nonpharmacological treatments helped to reduce burden and disability [12]. In adolescent patients, the inclusion of nonpharmacological approaches, such as nutraceuticals or behavioral techniques [13,14] as a support for traditional pharmacological therapies were suggested. Strong support for this conclusion came from the findings of two recent studies. The first reports the results of the CHAMP trial (Childhood and Adolescent Migraine Prevention), which compared amitriptyline and topiramate against placebo, and with each other, in patients with episodic and chronic migraine (excluding those with unrelenting headaches). The CHAMP trial was stopped early owing to futility, as no differences between amitriptyline, topiramate, or placebo with regard to headache frequency reduction and migraine-related disability were found over a 24-week period [15]. The results at three years basically confirm those observed at 24 months, with the patients maintaining the meaningful improvements achieved at the end of the trial, but with no difference between the treatment groups [16]. The second, a meta-analysis on the efficacy, safety, and acceptability of different pharmacological prophylactic treatments for pediatric migraine, showed that only propranolol and topiramate had higher efficacy than placebo in a short-term period, but not in the long-term [17]. Despite the fact that there were no safety issues raised with any of the drugs, the authors concluded that prophylactic medications should be carefully weighed against their potential harm, and that future research should identify nonpharmacological approaches.

Behavioral approaches are considered helpful for younger patients with HFEM and CM to manage pain, to reduce the number of analgesics and to reduce the use preventive medications [18,19,20]. Cognitive behavioral therapy (CBT) refers to a family of techniques aimed at helping patients to identify dysfunctional and recurrent thoughts to replace and integrate them with more functional beliefs [13]. In particular, CBT combined with a pharmacological approach, has been shown to reduce the burden of migraine by reducing the frequency and intensity of attacks and migraine-related disability [21]. CBT helps managing the psychological aspects related to pain, and is therefore helpful in reorganizing lifestyle habits allowing clinicians to monitor medication related issues, such as early treatment interruption, worries about drug side effects, and low compliance to the prophylactic therapy [18,19].

Mindfulness-based treatments have been applied in several clinical experiences in adults with pain and migraine, with encouraging results [22,23], as shown also in a review on psychological treatments for primary headache disorders [24], and with long term benefits similar to those obtained from traditional pharmacological therapies. For example, a fMRI recent study explained how mindfulness can change some cerebral patterns involved in pain and migraine pain, by influencing the activity of insula [25]. Insula is known to be an important cortical hub involved in the migraine phenomenon that processes many of the complex sensory and emotional aspects associated with the migraine condition and integrates many of the dynamic processes involved in migraine.

However, the clinical use of mindfulness in adolescents has not yet received the same attention as CBT. Some preliminary results in adolescents with chronic pain and headache disorders are encouraging [26,27,28,29,30]. One nonrandomized pilot study demonstrated acceptability and feasibility of a mindfulness-based treatment for adolescents with recurrent headaches [28]. Another study demonstrated how mindfulness was able to reduce depression in children and adolescents suffering from migraine associated with depression [29]. Another small pilot study [30] showed benefit with the use of mindfulness in young patients with headache without depression. This study highlighted the feasibility and the acceptability of a mindfulness-based protocol for adolescents with CM or HFEM.

These preliminary findings enable us to hypothesize that mindfulness may be beneficial in adolescents with CM or HFEM. In particular, we hypothesized that we would see a greater than 50% reduction in attacks with the use of mindfulness in at least half of our patients. The primary aim of this study was to examine the effect of a mindfulness-based intervention in adolescents aged 12–18 with CM or HFEM on 12-month headache frequency reduction used as primary endpoint. Secondary aims included the evaluation of mindfulness effects on medication intake, disability, anxiety, depression, catastrophizing, and caregivers’ burden.

## 2. Materials and Methods

### 2.1. Patients and Study Design

Adolescents aged 12–18 were consecutively enrolled at the Headache Center of the IRCCS Foundation Carlo Besta Neurological Institute in Milan (Italy) between November 2017 and November 2019. Follow-up concluded on January 2021 during regular clinical activity. Some patients were already in charge at our headache center while others were recruited on the occasion of their first visit. The period of enrolment was planned during the school period, i.e., no enrolment in the study after mid-June. Patients were invited to participate in the program after a discussion with their trusted neurologist and after informed consent was signed by patients and parents (a specific version of informed consent for parents and a specific version for patients were given).

Patients were diagnosed according to the criteria of the International Classification of Headache Disorders third version (ICHD-3), with CM (code 1.3 of ICHD-3) or episodic migraine without aura (code 1.1) at high frequency (HFEM). The latter included patients with 8–14 headache days per month in the last three months. As this was a pilot study, we did not conduct any statistical power calculation. We reasonably expected that over the two years of enrolment 30 to 40 patients could be included in the study, so the sample size is based on the available data.

Patients were excluded on the basis of the following criteria: major psychiatric disorders based on clinical history (e.g., personality disorders or psychotic disorders), psychotherapy in the previous 18 months or any previous experience of mindfulness or meditation approaches that might confound the effect of the proposed mindfulness-based treatment.

This was a single-arm open-label study. After enrolment, patients were followed-up for 12 months, with visits at 6 and 12 months (in which patients filled-in the whole protocol), and a phone contact at 3 months. This trial was performed in accordance with the declaration of Helsinki and was approved by the local Ethics Committee on December 2016 (protocol no. 35/2016; trial registration number on clinicaltrials.gov: NCT 04968093). The trial was conducted according to the original protocol. The analyses reported constitute the primary analyses of the MIND-KIDS pilot trial.

### 2.2. Procedure

Patients and parents were approached and asked to participate in the study on the occasion of a visit at our institute and asked to fill in the informed consent (signed by parents) and assent (signed by adolescents) in the case of acceptance. Those who accepted were invited to the preliminary session. Parents were given information about the rationale of the therapy, about the program and were provided with indications on how to help and to encourage home practice. Education is part of the treatment as the usual approach in our center. Patients and parents were provided with basic information on the occasion of the enrollment visit, whereas full information was provided at the preliminary session. Patients were educated and supported regarding a healthy lifestyle, introducing changes in habits when possible such as regular physical activity, avoiding skipping meals, hydration, maintaining a regular sleep/wake pattern with sleep at least 7–8 h per night.

The intervention was delivered at the Besta Institute in small groups (5–6 patients) in a silent room in which patients could stay in a comfortable position with a regular and adequate temperature and without any change other environmental conditions during the course of the treatment. The intervention was delivered once a week in the second half of the afternoon, to allow the participants to finish their school commitments, by an experienced neurologist (L.G.) and an experienced psychologist (El.G.), both with several years of experience in mindfulness practice and teaching in different clinical applications. The difference in expertise fields provided multiple points of view on the issue proposed during the sessions and specific information related to physical or psychological symptoms.

The intervention was aimed at reducing pain, increasing personal functioning and achieving skills to manage pain to continue everyday activities even in the presence of discomfort [20]. This intervention consisted of six weekly one-hour group sessions of a behavioral procedure held at the Besta Institute, one booster session after 15 days, and 10-min home self-practice, the latter done for the whole 12 months’ study duration.

We proposed an intervention implemented on the basis of the Mindfulness-Based-Stress-Reduction (MBSR) [31] and the Mindfulness-Based-Cognitive-Therapy (MBCT) [32] that have been used in several studies on patients with recurrent pain conditions [24]. We adapted these interventions with regard to the frequency and the duration of sessions to encourage participation and adherence to treatment considering and to the age group of the participants. A limited number of sessions was chosen to address fewer techniques, aimed at promoting mastery of a few techniques.

Each session included guided mindfulness-based meditation aimed at teaching and practicing to improve present moment awareness by directing sustained, nonjudgmental attention to the body. The meditation practice was performed without effort, and if distraction was present, patients were invited to gently pay attention to the assigned task, simply observing interfering thoughts in a neutral, nonjudgmental way. During the sessions, patients were asked to close their eyes and focus their attention on breathing so that they could concentrate on the present moment and on all the sensations. The sessions also included group discussions and sharing experiences on different topics such as posture education, breath use and control, guided body scans, work with sounds, tension release and guided imagery. Each session was conducted by a neurologist and a psychologist expert in mindfulness practice, and participants were invited to self-practice at home for at least 10-min/day by a standard recorded session provided by the psychologist. Psychological support was given by practical exercises to deal with stress and manage stressful situations by using metaphors and practical examples. Fifteen days from the end of the program, a booster session was performed to test the practice at home and to monitor clinical symptoms. Self-practice recommendation was made again at each follow-up examination, but no systematic evaluation was made on persistence of treatment.

During the 12-months study period no preventive medications were prescribed. Patients and their families could contact by phone or e-mail the clinicians in case of difficulty with symptoms management.

### 2.3. Measures

The research protocol included headache frequency (primary outcome measure) and medication intake, as well as a set of patient-reported outcome measures. Some of them addressing disability, anxiety, depression, and pain-related catastrophizing, were completed by patients. A measure of caregivers’ burden was completed by one of the two parents. Questionnaires and diaries were completed at baseline and at the two follow-up visits (6 and 12 months from baseline). All research material was completed in a paper/pencil way.

Headache diaries were used to address monthly headache frequency and medications intake. The latter included all available kinds of medications, with no distinction by compound.

The Pediatric Migraine Disability Assessment (PedMIDAS) [33] was used to address disability. The PedMIDAS is composed of six items addressing missed and partially missed school-days, missed and partially missed days for homework activities, and missed and partially missed days for leisure activities. The total PedMIDAS score is determined by the sum of item scores and four severity categories are established: 0–10 for no or minimal disability; 11–30 for light disability; 31–50 for moderate disability, and more than 50 for high disability.

Anxiety levels were measured using the Spielberger’s State-Trait Anxiety Inventory (STAI) for children [34] that is composed of 20 items, rated on a 1–4 scale, which evaluate state anxiety (i.e., temporary feelings at the time of a perceived threat; 0 = not at all—4 = very much) and 20 items evaluating trait anxiety (i.e., feelings of stress and worry experienced on a day to day basis; 0 = almost never—4 = almost always). The score range is between 20 and 80. Higher scores correspond to higher anxiety levels.

Depression level was measured with the Kovacs’s Children’s Depression Inventory (CDI) [35,36]. This questionnaire consists of 27 items evaluating different symptoms of depression, including mood disturbances, vegetative functions, social behavior, ability to feel good sensations, and self-esteem. The items investigate the influence of depression in specific contexts for young patients. Each item is scored on a 0–2 scale (0 = no problem; 1 = moderate problem; 2 = serious problem), and the total score range is 0–54. A total score higher than 19 indicates risk of depression.

Pain-related catastrophizing was measured with the Pain Catastrophizing Scale (PCS) [37]. This questionnaire addresses the concept of catastrophizing as it relates to pain, i.e., an exaggerated negative cognitive-affective response to anticipated or actual pain [38]. It is composed of three subscales each identifying specific dimensions: rumination, which refers to the constant thinking about pain (e.g., I can’t stop thinking about how much it hurts); magnification, which refers to the exaggeration of pain and of its consequences (e.g., I worry that something serious may happen); helplessness, which refers to the belief that there is no or limited possibility that pain may improve (e.g., there is nothing I can do to reduce the intensity of the pain). This measure is composed of 13 items, each rated from 0 = “not at all” to 4 = “all the time,” yielding total scores ranging from 0 to 52, with higher scores indicating higher levels of catastrophizing.

Caregivers’ burden was measured with the Caregiver Burden Inventory (CBI) [39]. The CBI consists of 24 items evaluated on a 0–4 scale (0 = not at all—4 = very much) that describe emotional reactions and difficulties related to the caregiving activity. The items assess issues related to the time required for assistance, the perception of being cut off from other activities and possibilities, fatigue, and the reduction of social activities and feelings towards the relative. The total score is linearly transformed on a 0–100 scale. Higher scores indicate greater burden.

### 2.4. Statistical Analysis

Descriptive statistics were used to present research data. Continuous variables were reported using means and 95% confidence intervals (95% CI), and categorical variables with frequencies and percentages. Suitability of data for parametric analysis was checked with Kolmogorov–Smirnov test and Q-Q plots.

We engaged only in complete analyses, i.e., no missing data replacement or imputation were made. Within-person ANOVA with Bonferroni post-hoc analysis was used to determine the significance of change between baseline and each time point of follow-up, i.e., 6 and 12 months after baseline. Cohen’s *d* was used as a measure of effect size (ES) for the change between each time point of assessment when the post hoc test showed significant variation. Coefficients around 0.3, 0.5 and around or above 0.8 respectively indicated small, medium and large change.

We also calculated the number of patients achieving 50% or higher reduction in headache frequency at both six and twelve months compared to baseline, and checked whether baseline differences existed between patients who achieved such reduction and those who did not, using an independent-sample *t*-test. Finally, we checked whether patients with the 6-month follow-up falling in the non-school period had a better outcome compared to those with the follow-up in the school period. Outcome was in these cases addressed as 50% or higher reduction achievement, and the Chi-Squared test was used to check this result.

For all analyses, statistical significance was set at the *p* < 0.05 level with two-tailed analysis. Data were analyzed with IBM SPSS statistics 26.0.

## 3. Results

A total of 37 patients were enrolled in the study: 35 were females and the average age was 15.2 (95% CI: 14.5; 15.9); 25 (67.6%) attended high school; regular physical activity and regular morning breakfast were reported by 29 (78.4%) patients each. Table 1 reports baseline information for headache frequency, medication intake and PROMs. Baseline monthly headache frequency was 21.1 (95% CI: 18.4; 23.7) and baseline monthly intake of medications was 7.3 (95% CI: 5.0; 9.6). A total of 29 out of 37 (78.4%) patients had CM, with a baseline monthly headache frequency of 24.5 (95% CI: 22.4; 26.7). Of them, 13 had migraine on a daily basis, and six were appointed medication overuse headache diagnosis. The first group session was held on November 2017 (follow-up closing on November 2018) and the last was held on January 2020 (follow-up closing on January 2021), for a total of seven groups. None of the patients interrupted the attendance to the mindfulness session.

Diaries with information on monthly headache frequency and medication intake were available for 35 patients over the entire follow-up period (the remaining two patients dropped out before the three-month phone contact, and we failed in obtaining any information), whereas the set of questionnaires were available for 33 patients only, because records of two patients had to be excluded due to incompleteness for the entire protocol. The remaining protocols were complete and attrition was relatively low (5.4% of loss for primary and 10.8% for secondary outcomes). All patients declared that over the 12-month period they regularly self-practiced at home for the suggested 10-min/day by a standard recorded session provided by the psychologist (i.e., none of them declared a break of the home practice over the year), but we did not assess this formally.

With regard to the primary endpoint, patients reported on average 21.3 (95% CI: 18.5; 24.1) headache days per month at baseline assessment, which dropped significantly to 9.6 (95% CI: 6.1; 13.1) with a mean reduction of 11.7 (95% CI: 7.3; 16.1) days per month. A total of 23 patients out of the 35 for who complete information on primary the endpoint was available (corresponding to 65.7%) achieved a reduction of headache frequency higher or equal to 50% at the 12-month follow-up. The same figure was 19 (54.3%) at the 12-month follow-up. No difference in any of the variables described was found at baseline between those who achieved, at both 6 and 12 months, the 50% or higher reduction in monthly headaches and those who did not. At the six-month follow-up in the non-school period for nine patients out of 35 (between mid-June and mid-July), five of these 9 adolescents (i.e., 55.6%) compared to 14 out of the 26 with follow-up in school period (i.e., 53.8%) achieved a 50% or higher reduction, and the Chi-Squared test was not significant.

Table 2 reports the results of the within-person ANOVA. For most variables (excluding anxiety state and caregivers’ burden) a significant improvement over the period was observed. Such improvement was detected between baseline and the 6-month follow-up and maintained up to the 12th month. Exceptions to this were the PedMIDAS and the trait subscale of the STAI, where the improvement was significant only between baseline and the 6-month follow-up, and the PCS-Magnification subscale, where the improvement was significant only between baseline and the 12-month follow-up. The magnitude of change was generally of medium to large size as shown by Cohen’s *d* ES, the only exception being CDI variation between baseline and 12-months, for which a small change was observed. No difference was detected between the 6-month and the 12-month follow-up in any of the variables.

## 4. Discussion

Our results show that the group of adolescents with CM or HFEM without aura enrolled in this study, who attended a seven-week mindfulness-based meditation group program and were not prescribed any pharmacological prophylaxis, underwent an improvement of both headache frequency and medication intake at both 6 and 12 months follow-up. They also improved in catastrophizing, symptoms of depression, trait anxiety, and disability and, for most of the variables, the improvement showed up between baseline and the 6 months follow-up, and were maintained up to the 12th month. Taken as a whole, our results suggest that group-based mindfulness intervention merits attention for the management of adolescents with CM and HFEM without aura.

The efficacy of a mindfulness-based program confirmed the results of Lovas and colleagues [27], in which an 8-week mindfulness-based intervention was carried out with adolescents, aged 14–17 years, with chronic idiopathic pain. In this study, the authors reported good acceptance of the program and significant improvements in pain intensity, somatic symptoms and disability, showing that mindfulness-based intervention could be useful in reducing chronic pain conditions in young people. This has been suggested in other studies [20,26,29].

With regard to patients’ adherence to the program, the six weekly sessions were attended almost completely by all patients, and none of them interrupted participation in the group sessions. In addition to the short program, the timing of the enrollment during the school period (and the achievement of the 12-months follow-up in that period) may have favored attendance at the treatment. None of the patients reported a break in home practice over the year. However, we did not formally investigate the patients’ persistence in home practice, i.e., we did not collect information on the frequency in which patients performed the suggested daily 10-min meditation.

Adherence to a mindfulness-based behavioral program was positively evaluated in the study of Hesse [28], that reported good acceptance of daily meditation practice both for clinical sessions and home practice. Hesse and colleagues enrolled 20 adolescents with “recurrent headaches”, defined as four or more headaches per month in the three months before enrollment, which were followed-up until the conclusion of the session, i.e., 8 weeks. The proposed program was adapted from the Mindful Schools curriculum for adolescents in a school context (18 sessions lasting 15 min each), for a clinic-based eight session lasting two hours each. The intervention was minimally tailored on headaches, and involved also depression and anxiety-related issues, as well as mindfulness-specific topics dealing with awareness of breath or mindfulness of sounds. This study did not demonstrate an effective decrease in migraine attacks, and no reduction in headache disability, while our study showed a reduction in the level of disability between baseline and six months. Hesse and colleagues hypothesized that the lack of reduction in disability was probably due to the severe headache burden, which may deserve more intensive and lasting behavioral and relaxation treatments to lead an effective reduction in headache-related disability [28].

The study by Kemper and colleagues [27], found that mindfulness intervention had a positive impact on reducing depressive symptoms in adolescents with recurrent migraine headache, and the same was shown by Hesse [28]. The results of our study confirm these findings, as our patients improved in Kovac’s CDI at both the 6 and 12-month follow-up and extended them to trait anxiety. The fact that an improvement at 6 and 12 months was observed for trait anxiety, but not for state anxiety, is of importance. In fact, trait anxiety reflects general anxiety symptom and not the way in which the patient feels in that specific moment which, in a population of adolescents, might be influenced by proximal factors such as school-related events happening contextually to the questionnaire’s completion. Any family or school-related event, in addition to the COVID-19 pandemic period, might influence trait anxiety. It can be presumed that positive and negative events were balanced, thus determining no variation in state anxiety score. Therefore, a significant improvement in trait anxiety scale might underlie a change in symptoms of anxiety resulting from mindfulness or headache frequency improvement.

Unfortunately, to the best of our knowledge, there is no investigation on the possible mechanisms of action of mindfulness-based treatment in adolescents with migraine. The results of a previous review suggested that headache improvement may be related, in part, to concurrent improvements in symptoms of anxiety and depression [19]. Such a hypothesis is plausible, as we also observed an improvement in anxiety and depression measures. In addition, we may hypothesize that the reduction of pain-catastrophizing, and therefore the greater control of pain-related events, may have played a role. A study conducted on adolescents with migraine and healthy controls showed that adolescents with migraine reported higher rumination and helplessness and a higher total PCS score. Authors hypothesized that these patients were characterized by sensory hypersensitivity that made them unable to manage overwhelming sensations similar to persons with pain catastrophizing. Therefore, the inefficiency of coping strategies to manage pain moments made both these individuals unable to adapt to the pain-related events [40,41]. Studies on adults also pointed out a role of neuro-inflammation [42], and of brain areas involved in pain representation (reduction in pain-related activation of the contralateral primary somatosensory cortex, and increased activation in the anterior cingulated cortex and anterior insula) [23,43]. However, it should be remembered that these considerations are drawn from adult populations and might be not entirely extended to pediatric patients.

Previous studies showed that pharmacological prophylaxis has little or no efficacy in the pediatric population of patients with migraine [15,16,17], and that future research should identify non-pharmacological approaches [17]. Addressing migraine early in this population is of importance in consideration of the fact that brain alterations begin early in youth with migraine, as showed in neuroimaging studies [44,45]. The result of our open-label trial supports the hypothesis that a combined approach that includes patient education and mindfulness-based programs can be very useful in populations of adolescents [20] and represent a valid alternative to pharmacological prophylaxis. The absence of a control group not receiving mindfulness does not enable us to determine how much of the change was due to the treatment and how much to placebo. An RCT comparing mindfulness against a “treatment as usual” condition is therefore needed. Such a study should include a clear investigation of the potential mechanisms of action of mindfulness.

Two notes on the timing of the study. First, several clinical check meetings took place during the lockdown period due to the COVID-19 pandemic. This could have had an impact not only on the home practice but also on the reduction in the expression of anxiety, depression and catastrophizing level. These data are in line with data from a multicenter Italian study that investigated changes in headache disorders during the COVID-19 lockdown among adolescents, which showed that the reduction of school effort and school-related anxiety was associated with an improvement in headache intensity and frequency [46]. Second, timing in relation to school activities should be considered. As our pilot study lasted 12 months and patients were enrolled during school period, all patients concluded the study during a school period. A minority of them (9 out of 35) had the 6 months follow-up at the beginning of summer holidays (mid-June to mid-July): however, in terms of achievement of the 50% or higher headache reduction, they did not perform better compared to the adolescents who completed the 6-month follow-up during school period.

Considering the behavioral nature and the commitment required in following the program, we believe that we included a sufficient number of patients who demonstrated good adherence to the treatment. This suggests that it is necessary to investigate and invest more in integrated and multidisciplinary approaches that can be of great support both for patients and their families. Indeed, these interventions help the patients manage and overcome the pain related difficulties in every-day life, reducing headache days and improving disability and coping strategies [20,47].

There are four main limitations of this study. First, although the selection criteria included patients with both HFEM and CM, the majority of enrolled patients had CM, and almost half of those with CM had migraine headaches on a daily basis. Such patients are representative of those that attend our institute but are only partly representative of the Italian population of adolescents with HFEM and CM. Thus, caution is warranted in the generalization of our results. Second, our study is based on a small sample of people. In addition, we acknowledge a selection bias. Most of the participants in our study were female, due to the fact that patients who attended our center and who agreed to participate in the study are mostly female. However, to our knowledge the few studies on the effects of a mindfulness-based intervention or a psychological intervention in general on adolescent patients do not focus on females only. For future studies, a more balanced sampling should be considered. Third, as this was an open-label study, we cannot determine how much of the effect at six and twelve months was due to the effect of the mindfulness-based treatment and how much to being included in a study, i.e., a placebo-like effect. A randomized trial including a “treatment as usual” control condition is therefore needed. Fourth, the delivery of the proposed mindfulness program did not include any control over patients’ adherence to daily home practice, which we did not investigate formally. Future studies focusing on adherence and persistence on mindfulness-based programs are needed to stress the impact of practice continuation on clinical outcome.

## 5. Conclusions

Our study represents one of the few studies that consider nonpharmacological therapies in adolescents with CM or HFEM, showing some feasibility in using these methods as part of the treatment in this patient population. As other studies have previously suggested, behavioral and mindfulness-based approaches can be of great support in reducing pain and disability in young patients [12,18,19,20,21], and represent a valid alternative to pharmacological prophylaxis which show little or no superiority to placebo [15,16,17]. The psychological aspects involved in the burden of headache in adolescents include different areas of life, not only of the patient themselves but also of the family members, so multidisciplinary care is necessary to improve the quality of life of all those involved [12].

## Figures and Tables

**Table 1:** Baseline description of enrolled patients.

	Mean (95% CI) N (N%)
Age	15.2 (14.6; 15.9)
Female Gender	35 (94.6%)
Disease duration, years	3.3 (2.4; 4.2)
Monthly headache frequency	21.1 (18.4; 23.7)
Monthly medication intake	7.3 (5.0; 9.6)
PedMIDAS	61.6 (49.1; 74.1)
STAI-State	33.4 (31.8; 35.1)
STAI-Trait	39.8 (37.6; 41.2)
CDI	14.1 (11.9; 16.4)
PCS total score	27.6 (24.9; 30.4)
PCS—Helplessness	12.8 (11.2; 14.3)
PCS—Rumination	12.3 (10.9; 13.7)
PCS—Magnification	2.6 (2.0; 3.2)
CBI	6.8 (3.7; 9.8)

Notes: All variables are reported as means and 95% CI, excluding gender. PedMIDAS, Pediatric Migraine Disability Assessment; STAI, State-Trait Anxiety Inventory; CDI, Kovacs’s Children’s Depression Inventory; PCS, Pain Catastrophizing Scale; CBI, Caregiver’s Burden Inventory; 95% CI, 95% Confidence Interval.

**Table 2:** Within-person ANOVA with Bonferroni post hoc test.

	Within-Person ANOVA Mean (95% CI for Mean)				Bonferroni Post Hoc Test Mean Difference (95% CI for Mean Difference) Cohen’s *d* Effect Size		
	Baseline	6 Months	12 Months	F (*p*)	Baseline—6 Months	Baseline—12 Months	6–12 Months
Monthly headache frequency	21.3	10.3	9.6	30.5	11	11.7	0.7
	(18.5; 24.1)	(7.1; 13.5)	(6.1; 13.1)	(*p* < 0.001)	(6.8; 15.2) ***	(7.3; 16.1) ***	(−3.4; 4.8)
					ES: 1.39	ES: 1.48	
Monthly medication intake	7.2	2	2.1	18.7	5.2	5.1	−0.1
	(4.8; 9.7)	(1.2; 2.9)	(0.9; 3.3)	(*p* < 0.001)	(2.4; 8.0) ***	(2.3; 8.0) ***	(−1.5; 1.4)
					ES: 0.75	ES: 0.74	
PedMIDAS	58.9	36.3	45.2	3.8	22.6	13.6	−9.0
	(46.3; 71.5)	(20.1; 52.5)	(28.7; 61.8)	(*p* = 0.027)	(2.5–42.7) *	(−8.9; 36.2)	(−28.6; 10.7)
					ES: 0.60		
STAI-State anxiety	32.8	31.5	32.5	0.8	–	–	–
	(31.1; 34.5)	(29.8; 33.3)	(30.6; 34.5)	(*p* = 0.447)			
STAI-Trait anxiety	39.3	35.6	36.2	5.1	3.7	3.1	−0.6
	(36.9; 41.7)	(33.6; 37.6)	(34.0; 38.4)	(*p* = 0.009)	(0.2; 7.2) *	(−0.3; 6.4)	(−3.1; 1.8)
					ES: 0.56		
CDI	13.2	9.2	10.7	9.5	4	2.5	−1.5
	(10.9; 15.4)	(7.2; 11.2)	(8.5; 12.9)	(*p* < 0.001)	(1.5; 6.4) **	(0.0; 4.9) *	(−3.6; 0.5)
					ES: 0.60	ES: 0.37	
PCS total score	27.8	19.2	16.9	23.6	8.6	10.9	2.3
	(24.9; 30.7)	(16.1; 22.4)	(13.5; 20.3)	(*p* < 0.001)	(3.9; 13.3) ***	(6.3; 15.5) ***	(−0.9; 5.5)
					ES: 1.06	ES: 1.35	
PCS–Helplessness	12.7	7.8	7.5	23.6	4.8	5.2	0.4
	(11.1; 14.3)	(6.4; 9.3)	(5.9; 9.1)	(*p* < 0.001)	(2.4; 7.3) ***	(2.8; 7.7) ***	(−0.9; 1.6)
					ES: 1.04	ES: 1.13	
PCS–Rumination	12.6	8.9	7.5	21.4	3.7	5.1	1.4
	(11.1; 14.1)	(7.5; 10.4)	(5.9; 9.1)	(*p* < 0.001)	(1.5; 5.8) ***	(2.9; 7.3) ***	(−0.3; 3.1)
					ES: 0.90	ES: 1.24	
PCS–Magnification	2.5	2	1.6	5.1	0.5	0.9	0.4
	(1.9; 3.2)	(1.4; 2.6)	(1.1; 2.2)	(*p* = 0.009)	(−0.2; 1.3)	(0.1: 1.7) *	(−0.2; 1.0)
						ES: 0.50	
CBI	7.3	5.3	5.4	1.2	–	–	–
	(3.9; 10.7)	(1.9; 8.7)	(1.8; 9.1)	(*p* = 0.311)			

Notes: PedMIDAS, Pediatric Migraine Disability Assessment; STAI, State-Trait Anxiety Inventory; CDI, Kovacs’s Children’s Depression Inventory; PCS, Pain Catastrophizing Scale; CBI, Caregiver’s Burden Inventory; 95% CI, 95% Confidence Interval; ES, Effect Size. For Bonferroni post-hoc analysis, * *p* < 0.05, ** *p* < 0.01, *** *p* < 0.001.

## Data Availability

The data presented in this study are available on request from the corresponding author.
